# Impact of Variation at the FTO Locus on Milk Fat Yield in Holstein Dairy Cattle

**DOI:** 10.1371/journal.pone.0063406

**Published:** 2013-05-15

**Authors:** Lea G. Zielke, Ralf H. Bortfeldt, Monika Reissmann, Jens Tetens, Georg Thaller, Gudrun A. Brockmann

**Affiliations:** 1 Department of Crop and Animal Sciences, Humboldt-University Berlin, Berlin, Germany; 2 Institute of Animal Breeding and Husbandry, Christian Albert University Kiel, Kiel, Germany; The Children's Hospital of Philadelphia, United States of America

## Abstract

This study explores the biological role of the Fat Mass and Obesity associated (FTO) gene locus on milk composition in German Holstein cattle. Since FTO controls energy homeostasis and expenditure and the FTO locus has repeatedly shown association with obesity in human studies, we tested FTO as a candidate gene in particular for milk fat yield, which represents a high amount of energy secreted during lactation. The study was performed on 2,402 bulls and 860 cows where dense milk composition data were available. Genetic information was taken from a 2 Mb region around FTO. Five SNPs and two haplotype blocks in a 725 kb region covering FTO and the neighboring genes RPGRIP1L, U6ATAC, and 5 S rRNA were associated with milk fat yield and also affected protein yield in the same direction. Interestingly, higher frequency SNP alleles and haplotypes within the FTO gene increased milk fat and protein yields by up to 2.8 and 2.2 kg per lactation, respectively, while the most frequent haplotype in the upstream block covering exon 1 of FTO to exon 15 of RPGRIP1L had opposite effects with lower fat and milk yield. Both haplotype blocks were also significant in cows. The loci accounted for about 1% of the corresponding trait variance in the population. The association signals not only provided evidence for at least two causative mutations in the FTO locus with a functional effect on milk but also milk protein yield. The pleiotropic effects suggest a biological function on the usage of energy resources and the control of energy balance rather than directly affecting fat and protein synthesis. The identified effect of the obesity gene locus on milk energy content suggests an impact on infant nutrition by breast feeding in humans.

## Introduction

Genome-wide association studies (GWAS) have repeatedly shown that the Fat Mass and Obesity associated (FTO) gene region is associated with differences in human body mass index (BMI), predisposition to type II diabetes and obesity [1]–[5]. The effects observed for the FTO region were about 3 kg of the body mass or 0.39 kg/m^2^ of the BMI for humans that are homozygous for the risk allele [2], [6]. Therefore, the FTO region represents a small effect locus contributing to a complex trait [7]. Further knowledge of phenotypic effects of such loci on additional traits, which is challenging to measure in humans, would be beneficial. Since the FTO protein is conserved with a sequence identity of over 85% among humans, mice, cattle, sheep, dogs and horses [8], it is conceivable that it shares similar functions among all vertebrates.

Experiments in mouse and rat models confirmed the influence of FTO in the central control of energy homeostasis and the control of energy expenditure [8], [9]. For example, FTO deficiency in knock-out mice led to postnatal growth retardation accompanied by a significant reduction of adipose tissue and lean body mass [9]. Furthermore, it was shown that the FTO protein shares sequence motifs with the Fe(II)-and 2-oxoglutarate-dependent oxygenases [10]. Therefore, it was assumed that FTO has an important role in DNA repair and post-translational modifications. Additional experiments have identified that FTO signalizes cellular availability of oxygen, is functionally involved in fatty acid metabolism and energy homeostasis, and has a role in the catalysis of nucleic acid demethylation [11].

Body weight regulation was suggested to arise from activity of the FTO protein in brain regions that control food intake since different transcript amounts were found according to food intake and deprivation [8]. Experiments on pigs and sheep showed a significantly higher expression of FTO in brain regions such as cortex, hippocampus and hypothalamus [12], [13]. Additional studies in pigs provided evidence that FTO was associated with intramuscular fat deposition and average daily gain [14], [15].

While human GWAS reported FTO as the major candidate gene for the obesity associated genomic region, additional significant SNPs were located in the close neighborhood of FTO, in particular, in the RPGRIP1-like (RPGRIP1L) gene [1]–[5]. This gene encodes a protein with a conserved C2-domain often found in calcium dependent membrane proteins, which bind phospholipids, inositol polyphosphates, and intracellular proteins [16]. Experiments with primary human pre-adipocytes isolated from adipose tissue showed that RPGRIP1L might be involved in adipogenic differentiation and has a potential role in the insulin regulated adipocyte metabolism [16].

Although a number of cellular, molecular and genetic studies have been performed with FTO, its functions and effects are far from being understood. Even less is known about RPGRIP1L. Considering that the FTO gene including its linked genomic neighborhood affects fat deposition in humans, the question arises, whether this gene region also affects the amount of fat delivered in milk during lactation. If the FTO locus does not only affect fat synthesis but is also involved in the regulation of energy balance, we would also expect additional effects on other milk components. This would not only extend our current knowledge on the FTO region but also have an impact on maternal genotype driven effects on infant nutrition via breast feeding.

The objective of this study was to explore the biological role of the FTO locus as a functional candidate on milk composition, in particular fat yield. Since effects on milk composition are difficult to test in humans, we performed an association study between genetic variation in the FTO region and milk composition traits in German Holstein cattle. The advantage of dairy cattle is the availability of monthly records of milk yield and composition during the whole lactation period. Estimated breeding values (EBV) of bulls based upon the production performance data of all their daughters are highly reliable and still more accurate than production data of individual cows. Our analyses provided significant association of five SNPs and two haplotype blocks, which are either directly located in the FTO gene or in close proximity.

## Results

### Variation in the FTO Gene Region

**Figure 1:**
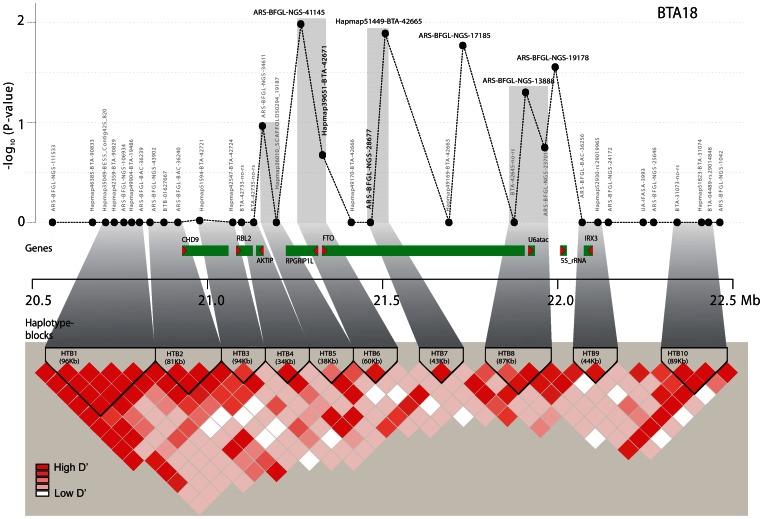
Gene and haplotype block structure of the 2 Mb FTO region and LD heat map. In the upper part, negative log_10_ p-values of the regression model 4 (Table 1) are shown for SNPs within the 2 Mb target region of FTO. All genes of the region are highlighted in dark grey, with an arrow head pointing in the direction of transcription. The lower part shows the pairwise D’ for the FTO region calculated with Haploview. Haplotype blocks are indicated with black triangles.

In the analyzed 2 Mb region surrounding the FTO gene, eight genes and 36 SNPs are located. Seven intronic SNPs lie directly in the FTO gene (Figure 1, Table S1 in File S1). We identified ten haplotype blocks (HTB) in the 2 Mb FTO region (Figure 1, Table S2 in File S1). The largest block HTB1 spans 96 kb and consists of five SNPs with an average D’ of 0.97 as a measure for linkage disequilibrium (LD). Six haplotype blocks consist of two SNPs, spanning 34 to 94 kb with an average D’ between 0.59 and 0.98. Haplotype block HTB7 is located within the FTO gene, whereas haplotype blocks HTB6 and HTB8 have one SNP in the FTO gene and the other one in the RPGRIP1L gene or in the intergenic region between U6ATAC and 5 S rRNA.

### SNP Associations with Milk Fat Yield in the Bull Population

Five out of 36 SNPs were significantly associated (p<0.05) with the average EBV for milk fat yield over the first three lactations when the most stringent model 4 was applied accounting for population stratification and the known major gene effect of DGAT1 [17] (Table 1, Table 2, Table S1 in File S1). For clarity, we numbered the five significant SNPs and two more SNPs contributing to significant haplotypes from 1 to 7 (Table 2, Table 3). Of the five significant SNPs, which are all located in a 725 kb region, two are located in introns 6 and 8 of the FTO gene (Hapmap51149-BTA-42665 (SNP4), ARS-BFGL-NGS-17185 (SNP5)), one SNP lies 54 kb upstream of FTO (ARS-BFGL-NGS-41145 (SNP1) in exon 15 of the RPGRIP1L gene, and two SNPs lie 2.7 and 87 kb downstream (ARS-BFGL­NGS-13888 (SNP6), ARS-BFGL-NGS-19178 (SNP7)) of FTO close to the genes U6ATAC (8.81 kb upstream) and 5 S rRNA (12.23 kb downstream).

**Table 1:** Association models tested in this study.

Model	Description
1	Y = β_0_+β_1_X+ε
2	Y = β_0_+β_1_X+β_3_D+ε
3	Y = β_0_+β_1_X+β_2_Z+ε
4	Y = β_0_+β_1_X+β_2_Z+β_3_D+ε

Four different models (1–4) with increasing stringency criteria as tested in the association analyses. Y = EBVs for milk fat yield, X = matrix of coded alleles, β = regression coefficient, Z = covariance matrix from multidimensional scaling, D = covariance vector of DGAT1 alleles.

**Table 2:** Results of SNP association analysis with milk fat yield (FY) (estimated breeding values (EBV) in bulls, yield deviations (YD) in cows).

							Regression model					
Popu-lation	SNP number	SNP ID	Position	A1	A2	f_A1_	No Adjustment	DGAT1	Population structure	DGAT1+ Population structure	β	*a*
							Model 1	Model 2	Model 3	Model 4	Model 4	Model 4
A	1	*ARS-BFGL-NGS-41145*	21267130	G	A	0.45	n.s.	n.s.	0.0413	0.0103	2.52	1.46
	2	*Hapmap39651-BTA-42671*	21327138	C	A	0.41	n.s.	0.0404	n.s.	n.s.	1.95	1.49
	3	*ARS-BFGL-NGS-28677*	21464728	A	G	0.41	n.s.	n.s.	n.s.	n.s.	0.72	1.98
	4	*Hapmap51449-BTA-42665*	21508221	G	A	0.26	0.0083	0.0002	0.0563	0.0129	−2.77	2.86
	5	*ARS-BFGL-NGS-17185*	21729770	G	A	0.35	0.0088	8.06e–05	n.s.	0.0172	2.53	2.30
	6	*ARS-BFGL-NGS-13888*	21907404	G	A	0.40	n.s.	n.s.	n.s.	0.0496	−2.20	1.62
	7	*ARS-BFGL-NGS-19178*	21991700	A	G	0.28	n.s.	0.0629	0.0666	0.0279	−2.61	2.64
B	1	*ARS-BFGL-NGS-41145*		G	A	0.45	0.0414	n.s.	n.s.	n.s.	2.76	5.53
	2	*Hapmap39651-BTA-42671*		C	A	0.43	0.0030	0.0105	0.0379	n.s.	3.53	6.03
	3	*ARS-BFGL-NGS-28677*		A	G	0.47	n.s.	n.s.	n.s.	n.s.	−2.37	1.39
	4	*Hapmap51449-BTA-42665*		G	A	0.24	n.s.	n.s.	n.s.	n.s.	−2.83	5.83
	5	*ARS-BFGL-NGS-17185*		G	A	0.34	n.s.	n.s.	n.s.	n.s.	0.89	0.05

Shown are p-values for SNPs within the 2 Mb FTO region on chromosome BTA18 that are significantly associated with the average EBV for fat yield (FY) over lactations one to three after applying model 4 in the bull (**A**) and cow population (**B**). The SNPs numbered 1 to 5 are identical in bulls and cows. SNPs 1, 4 and 5 were genotyped in cows as most significant SNPs in bulls. SNPs 2 and 3 were not significant in bulls in model 4 but were genotyped in cows to derive haplotypes in HTB 6 and 7 (see **Table 3**). P-values for all four models (Table 1) are shown for comparison. Positions refer to the Btau 4.2 assembly. f_A1_ = population frequency of the minor allele; α = additive effect, β = effect size, giving the per minor allele change of the trait; α and β are given in kilogram fat as EBV units in bulls and YD units in cows. Since the correction for environmental effects differs between EBV and YD, the units are not identical. P-values >0.1 are labeled “n.s.”.

**Table 3:** Results of haplotype association analysis with milk fat yield (FY) (estimated breeding values (EBV) in bulls, yield deviations (YD) in cows).

						Regression model							
Popu-lation	SNP number	SNP ID	Block ID	Haplotype	f_hpt_	No Adjustment	DGAT1	Population structure	DGAT1+ Population structure	β		µ±se (FY)	
						Model 1	Model 2	Model 3	Model 4	Model 4			
A	1	*ARS-BFGL-NGS-41145*	HTB6	AA	0.52	n.s.	0.0081	0.0058	0.0058	−2.62		19.10±0.47	
	2	*Hapmap39651-BTA-42671*		GC	0.37	n.s.	n.s.	n.s.	n.s.	1.90		20.61±0.58	
				GA	0.08	n.s.	n.s.	n.s.	n.s.	2.65		21.14±1.22	
				AC	0.03	n.s.	n.s.	n.s.	n.s.	1.22		22.60±1.93	
A	3	*ARS-BFGL-NGS-28677*	HTB7	AA	0.41	n.s.	n.s.	n.s.	n.s.	1.65		20.98±0.54	
	4	*Hapmap51449-BTA-42665*		GA	0.34	n.s.	n.s.	n.s.	n.s.	0.69		20.25±0.62	
				GG	0.26	0.0082	0.0002	0.0129	0.0129	−2.79		17.82±0.67	
				AG	0.0009	n.s.	n.s.	n.s.	n.s.	–		21.70±13.27	
B	1	*ARS-BFGL-NGS-41145*	HTB6	AA	0.46	0.0005	0.0012	0.0293	0.0897	−4.49		0.75±1.53	
	2	*Hapmap39651-BTA-42671*		GC	0.34	0.0161	0.0419	n.s.	n.s.	3.19		8.20±1.70	
				GA	0.11	n.s.	n.s.	n.s.	n.s.	4.71		6.57±3.35	
				AC	0.09	n.s.	0.0403	n.s.	n.s.	1.02		9.55±3.49	
B	3	*ARS-BFGL-NGS-28677*	HTB7	AA	0.44	n.s.	n.s.	n.s.	n.s.	−2.96		4.19±1.58	
	4	*Hapmap51449-BTA-42665*		GA	0.32	0.0092	0.0054	0.0182	0.0210	6.86		8.61±1.82	
				GG	0.21	0.0432	n.s.	n.s.	n.s.	−1.86		0.59±2.18	
				AG	0.03	n.s.	n.s.	n.s.	n.s.	−5.93		0.81±5.69	

Shown are p-values for haplotypes within the 2 Mb FTO region on chromosome BTA18 that are signifcantly associated with the mean EBV for fat yield (FY) over the first three lactations after applying model 4 in the bull-(**A**) and cow population (**B**). P-values for all four models (Table 1) are shown for comparison. Positions refer to the Btau 4.2 assembly. β = effect size (regression coefficient) giving the per haplotype change of the trait. µ ± se denotes the phenotypic mean with standard error of the haplotype class based on EBVs in bulls and YDs in cows. β and µ are given in kilogram fat as EBV units in bulls and YD units in cows. Since the correction for environmental effects differs between EBV and YD, the units are not identical. f_hpt_ = haplotype population frequency. P-values >0.1 are labeled “n.s.”.

**Figure 2:**
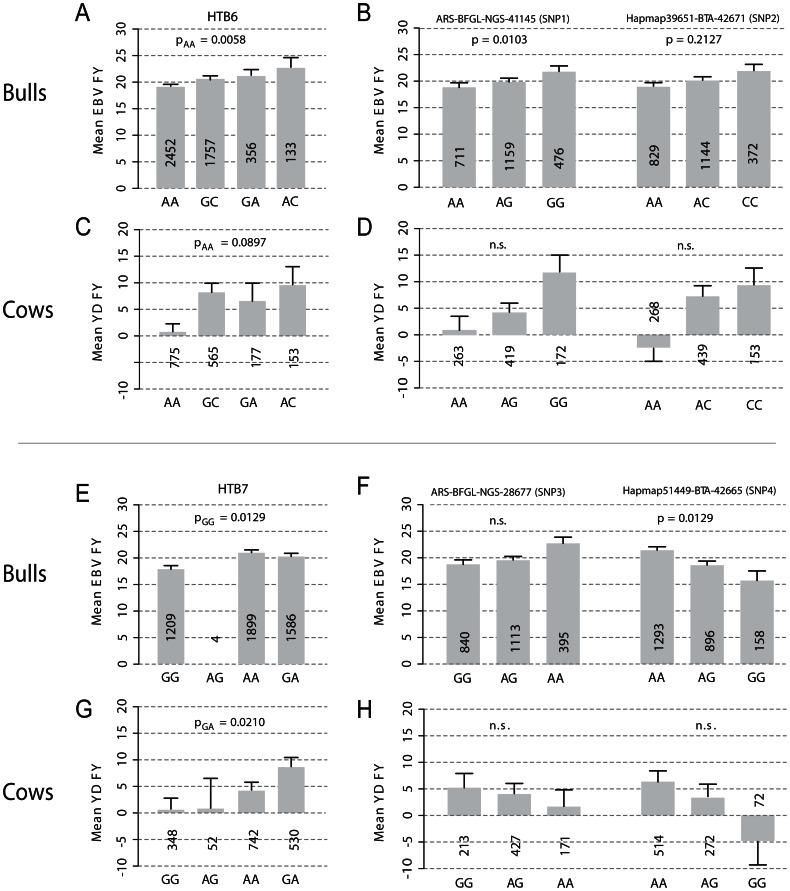
Effect plots for significant haplotype blocks (A, C, E, G) and SNPs building these blocks (B, D, F, H) in the bull (A, B, E, F) and cow population (C, D, G, H). Haplotype block HTB6 consists of the SNPs ARS-BFGL-NGS-41145 (SNP1) and Hapmap39651­BTA-42671 (SNP2); HTB7 of ARS-BFGL-NGS-28677 (SNP3) and Hapmap51149­BTA-42665 (SNP4). Shown are mean and standard error for estimated breeding values (EBVs) and yield deviations (YDs) for milk fat yield (FY) over lactations 1 to 3 in bulls and cows, respectively. EBVs of bulls represent the milk production of daughters, YD of cows refer to own milk production data. Numbers represent counts of observed genotypes and haplotypes, respectively. Haplotypes are ordered according to their frequency in bulls, beginning with the most frequent haplotype. The frequency of haplotype AG in HTB7 was below 1% in the bull population and was not considered in the association study. P-values are given for regression model 4 (Table 1) considering population stratification and DGAT1 effects. For significantly associated SNPs and haplotypes, Bonferroni corrected p-values of the regression model are given for the bull population; for the cow population p-values of the Tukey Kramer test are given for SNPs and p-values of the mixed model are given for the haplotypes (Tables 2 and 3). P_AA_ and P_AG_ are p-values for the haplotypes AA and AG in HTB6 and HTB7, respectively, indicating that these haplotypes differ significantly from the group of other haplotypes in the corresponding haplotype block (Table 3).

The SNP ARS-BFGL-NGS-41145 (SNP1), which is located in the RPGRIP1L gene and showed the lowest p-value (p = 0.0103) in both models with population stratification, accounted for a minor allele effect of 2.52 kg milk fat over the first three lactations and an average difference of 3.10 kg between the low fat homozygous class AA and the high fat class GG (Table 2A). The high fat genotype GG occurred with the lowest frequency (0.20) (Figure 2B). The SNP Hapmap51149BTA-42665 (SNP4) in intron 6 of the FTO gene (Figure 2F) had the highest effect size for milk fat yield and was significant in all four models (p = 0.0129 in model 4), but the direction of effect of the minor allele was opposite to SNP ARS-BFGL-NGS-41145 (SNP1) (Table 2A). Bulls of the most frequent genotype class AA (0.55) had a mean EBV for milk fat yield of 21.41 kg, while the mean EBV in the lowest frequent genotype class GG (0.07) was 15.70 kg and of heterozygous bulls 18.59 kg (Table S3 in File S1). The second significant SNP in the FTO gene (p = 0.0172) was ARS-BFGL-NGS-17185 (SNP5) in intron 8. This SNP had a positive minor allele effect size of 2.53 kg for milk fat yield. The two significant SNPs downstream of FTO (SNP6, SNP7) showed similar direction of effect as SNP4 in the FTO gene (Table 2A).

**Figure 3:**
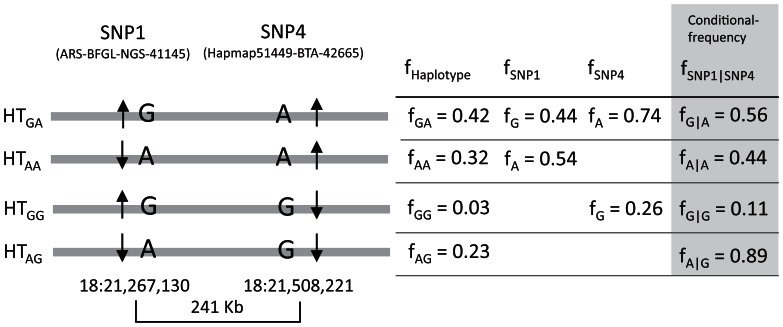
Haplotypes between ARS-BFGL-NGS-41145 (SNP1) and Hapmap51449-BTA-42665 (SNP4). Both SNPs are associated with milk fat yield, but occur with different allele frequency distributions and direction of effect. D’ for the linkage between the two SNPs is 0.759. Frequencies of observed haplotypes as well as total and conditional allele frequencies of SNP1 and SNP4 are given. Arrows indicate the direction of effect on milk fat yield.

Since the significant SNPs had different direction of effect, we had a closer look at the pair-wise linkage disequilibrium between these SNPs (Figure 1, Table S6 in File S1). SNP1 and SNP4, located in HTB6 and HTB7, respectively, were also in high LD with a D’ value of 0.76. The high frequency milk fat increasing allele A of SNP4 (frequency of 0.74) was linked with the high fat allele A of SNP1 at a frequency of 0.56 and with the low fat allele G at a frequency of 0.44, while the minor and low fat allele G of SNP4 was almost entirely linked with SNP1 allele A (Table S6 in File S1). This suggests that the mutation linked to SNP1 occurred on the strand of allele A of SNP4 (Figure 3).

### Haplotype Association Analysis with Milk Fat Yield in the Bull Population

Haplotypes in the blocks HTB6, spanning a region from exons 1 to 15 in RPGRIP1L and intron 1 in FTO (p = 0.0058 for haplotype AA) and HTB7, which is located directly in the FTO gene (p = 0.0129 for haplotype GG), were significantly associated with the EBV for milk fat yield in each lactation and the average of them (Table 3A). Models 3 and 4, which both considered the population stratification but differed in accounting for DGAT1 had the same significant p-values. Each of the haplotype blocks consists of two SNPs with one of them being significant: ARS-BFGL-NGS-41145 (SNP1) in HTB6 and Hapmap51449-BTA-42665 (SNP4) in HTB7. In haplotype block HTB6, the least frequent haplotype AC (0.03) showed the highest, whereas the most frequent haplotype AA (0.52) showed the lowest phenotypic mean for milk fat yield. The difference between these two haplotypes was 3.50 kg milk fat (Figure 2A). In contrast, the least frequent haplotype GG (0.26) in HTB7 showed the lowest mean (17.82 kg) being significantly lower than the means of the haplotypes GA (20.25 kg, p = 0.0205) and AA (20.98 kg, p = 0.0009), respectively (Figure 2G).

### SNP Association Analysis with Milk Fat Yield in the Cow Population

Allele frequencies in the cow population were similar to the bull population for all SNPs. In cows, none of the SNPs that previously showed associations in the bull population was significant (Table 2). Only SNP Hapmap39651BTA-42671 (SNP2), located in HTB6, was significantly associated with yield deviations of milk fat yield, if model 3 was applied accounting for population stratification but not for DGAT1 (p = 0.0379). The homozygous genotype classes AA and CC of this SNP (p = 0.0045) differed by 11.82 kg average milk fat in the first three lactations (Table S3 in File S1). In respect to lactation effects, Hapmap39651-BTA-42671 (SNP2) was also significant for lactation 2 even under model 4 (p = 0.0448). In comparison, in model 3, taking only the father as a random effect and omitting DGAT1 as a fixed effect, this SNP showed significant associations in lactations 2 and 3 (data not shown).

### Haplotype Association Analysis with Milk Fat Yield in the Cow Population

The haplotype blocks HTB6 and HTB7 that were significant in bulls were also significantly associated with milk fat yield in cows (Table 3B). The haplotypes AA in HTB6 and GA in HTB7 were significant in all models at a p-value <0.05, except haplotype AA in HTB6, which was only modestly significant (p = 0.0897) in model 4 after fitting both the sire and the DGAT1 effect.

In HTB6, the haplotype AA, which was significant in the bull population, was also significant in the cow population. Consistent with the bull population, the haplotypes with the highest and lowest mean were AC and AA, respectively (Figure 2C).

In HTB7, different haplotypes were significant in cows and bulls. In cows, the significant haplotype GA had the highest mean and about twice the amount of milk fat yield deviation (8.61±1.82 kg) compared to the most frequent haplotype AA (4.19±1.58 kg) (Figure 2G). The high difference between the haplotypes in cows was not observable in the bull population, although the direction of effect was the same.

### Effects on Other Milk Composition Traits

**Figure 4:**
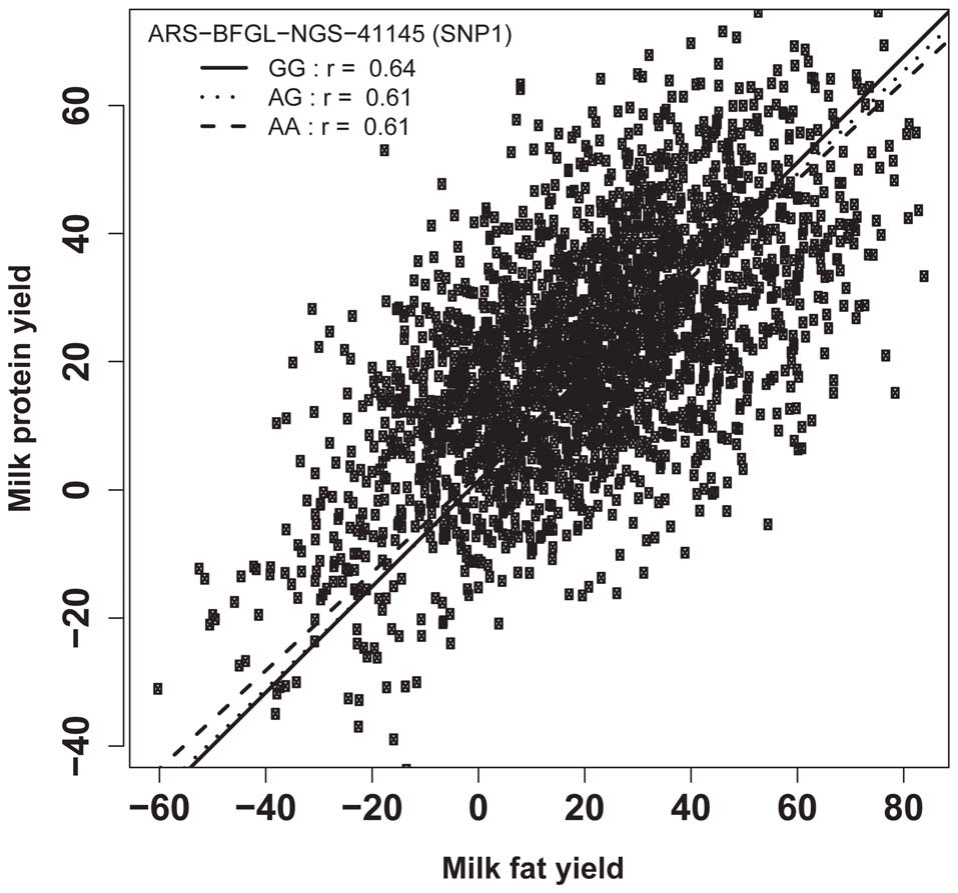
Relationship of milk fat yield to milk protein yield for SNP ARS-BFGL-NGS-41145 (SNP1) in the bull population (Pearson correlation). The solid line is the regression of fat yield on protein yield in the GG genotype class, the dotted line in the AG, and the dashed line in the AA genotype class.

In bulls, the two significant SNPs with the lowest p-values that were associated with the EBV for milk fat yield also showed a significant association with other milk traits (Table S4 in File S1). The SNP ARS-BFGL-NGS-41145 (SNP1) in haplotype block HTB6 was significantly associated with the average milk and protein yield for all lactations. Additional associations with protein yield were found for SNP Hapmap51449–42665 (SNP4) in haplotype block HTB7, where the homozygous genotype of the minor allele (allele frequency 0.26) showed the lowest mean for all yield traits. The correlation coefficients between milk fat yield and milk protein yield conditioned for the three genotype classes at SNP1 were >0.60 (p<10^−15^) (Figure 4).

In addition to SNPs, the most frequent haplotype AA of HTB6 was significant for milk and protein yield in bulls (Table S5 in File S1). The effects of this haplotype were negative for all traits. Additionally, haplotype GA of the same haplotype block showed a significant positive association with milk yield in bulls. In HTB7, haplotype GG had a significant negative impact on protein yield in bulls.

## Discussion

### Effects of the FTO Locus on Milk Fat Yield and Other Milk Composition Traits

In the current study, we tested the biological function of a 2 Mb region of the FTO locus on milk composition in German Holstein dairy cattle. Evidence for association of this locus with milk fat yield was provided by analyses of estimated breeding values of 2,402 bulls and yield deviations of 860 cows for the average milk fat yield in lactations 1 to 3. The breeding values of bulls are highly precise phenotypes as they are based on the milk production of their daughters, while yield deviations of cows are own performance data. Significant genetic effects on milk fat yield were identified within a 725 kb region for five SNPs in the bull population and two haplotype blocks in the bull and the cow population. This region encompassed the FTO gene, part of the neighboring upstream gene RPGRIP1L, and the downstream genes U6ATAC and 5 S rRNA.

The most significant evidence for association of the FTO locus with milk fat yield came from associated haplotypes in the blocks HTB6 and HTB7, which covered exons 1 to 15 of RPGRIP1L and exon 1 of the FTO gene and exons 4 to 6 of FTO, respectively. This also provided evidence that the haplotypes captured more genetic variation than the genotyped SNPs alone. In haplotype block HTB6, not only the same haplotype AA was significant in bulls and cows, but the magnitude and direction of effect were the same as well. In both populations, the most frequent haplotype AA was associated with the lowest milk fat yield. In haplotype block HTB7, different haplotypes were significant in the bull (GG) and in the cow (GA) population. Nevertheless, the direction of effect of all haplotypes in this block was similar.

The picture was not that clear, if we looked at associated SNPs alone. While five SNPs were significant in the bull population, there was just one SNP in cows, which was not even significant in bulls. Since the direction of effect was the same for all significant SNPs in cows and bulls, the differences in p-values mainly result from the higher population size (2,402 bulls vs. 860 cows) and accuracy of phenotypes in bulls (EBVs vs. own performance data), differences in the population structure and accounting for it (IBS clusters vs. random father), and random sampling affecting allele frequencies and LD between markers [3], [18].

Interestingly, our data provided evidence that the two most significant SNPs 1 and 4 and the haplotypes to which these SNPs contributed were not only associated with milk fat yield but also with milk protein yield. Although EBVs for lactose yield were not available, the SNP upstream of FTO in the RPGRIP1L gene is an indication for association with lactose yield, as this SNP influenced milk yield, which is mainly regulated by the osmotic pressure of lactose [19]. Since the direction of effect for all yield traits was the same at every locus, pleiotropic gene action is likely.

Our findings suggest that the FTO region not only regulates milk fat yield, but also the total energy content of milk. With regard to GWAS in humans, the FTO region has been repeatedly associated with body mass index and obesity. However, studies with lean mass have not been performed. To further test the pleiotropic effects of the FTO region, the analysis of traits characterizing body composition would be of interest. But body mass measurement of dairy cattle is not a matter of routine under production conditions.

The effects of the genetic variation in the FTO region accounted for about 1% of the corresponding traits variance in the analyzed cattle population. Even if the effect is small, it seems to be consistent across species and deserves more attention as a factor contributing to complex traits, which are expected to be formed by small effects of many loci [7].

### Genetic Architecture of the FTO Locus

Frequencies and direction of effect showed differences between haplotype blocks HTB6 and HTB7. In HTB7, located in the FTO gene, we observed higher frequencies of the high yield haplotypes, while in HTB6, which covers partially RPGRIP1L and the beginning of FTO, high frequencies were found for the low yield haplotypes. The opposite direction of effect of the major alleles was best demonstrated by SNP1 and SNP4, contributing to HTB6 and HTB7, respectively. The frequency of the high performing allele of SNP4 (0.75) was considerably higher than of SNP1 (0.45). Therefore, we suggest a causative mutation in the region linked to SNP4 in the middle of the FTO gene which has been under selection pressure for high yield traits and another mutation linked to SNP1. Since German Holstein cattle have been under selection for high milk production during the last decades, we would expect increased allele frequencies at loci that have positive effects on production traits like yield traits [20]. However, the neighboring upstream region of HTB6 shows more balanced frequencies of the high and low fat alleles. Thus, we conclude that the mutation at SNP1 occurred on the chromosomal strand of allele A of SNP4 before selecting for milk traits.

### The Potential Biological Role of the FTO Locus

The different effects of the two haplotype blocks HTB6 and HTB7 in combination with a shift in allele frequency distribution, provide evidence for two or more mutations at the FTO locus that affect the traits. These findings also indicate that not only FTO but also RPGRIP1L contributes to milk composition. In mouse and human tissues similar expression patterns were found for FTO and RPGRIP1L, suggesting a co-expression of these two genes [21]. However, this co-regulation has been questioned since feeding experiments on mice demonstrated that FTO was down regulated during fastening whereas RPGRIP1L was not [21]. Our data support that FTO and RPGRIP1L are not co-regulated. In addition, pleiotropic genetic effects of different mutations can be assumed, because the direction of effect of a SNP was always the same for all traits. This would imply that the observed genetic effects control the fat and protein metabolism or generally energy homeostasis and energy partitioning.

Although no attention has been paid to the influence of the FTO locus on protein synthesis or lean mass in human obesity studies, our observation that milk fat and protein yield are affected is in agreement with the phenotype of FTO knock-out mice. They were described as growth retarded with reduced fat and lean mass, demonstrating a pleiotropic effect on both [9]. Since the FTO protein itself has an alpha-ketoglutarate binding site and dioxygenase activity, it can be assumed that the molecular action of FTO is at least partly due to the enzymatic regulation of carnitine biosynthesis, which is an essential metabolite in eukaryotes required for fatty acid oxidation [22]. The carnitine biosynthesis pathway links the protein with the fatty acid metabolism. An association between weakness and carnitine deficiency has previously been described [23]. The coordinated control of yield traits mirrors the secretion of a high or low amount of energy via milk and, thus, reﬂects the control of energy partitioning as a whole instead of direct effects on metabolic pathways.

Downstream of FTO, two SNPs adjacent to the genes U6ATAC and 5 S rRNA were associated with milk fat yield in our study. These genes are transcribed into non-coding RNAs, which are components of the minor spliceosome and the ribosome. As such they are key elements of transcription and protein synthesis. For this reason, they cannot be ruled out as potential units that directly or indirectly affect milk protein and fat synthesis.

In summary, our study in dairy cattle provides evidence that the obesity-associated FTO gene region accounts for variation in milk fat yield. For the first time, we show that the region does not only control fat but also protein yield and that both milk composition traits are regulated in the same direction. Therefore, we suggest that the FTO gene region controls the energy amount secreted during lactation. The position of the associated haplotype blocks and SNPs, their direction of effect and allele frequency distribution detected in our cattle study suggest that at least two causative variants account for differences between genotype classes. These mutations most likely underlie different selection pressure for production traits. In turn, this indicates different biological functions of the involved gene variants with respect to control and regulation of fat and protein metabolic pathways and in regard to maintaining energy homeostasis and controlling energy partitioning. Besides FTO, the neighboring upstream gene RPGRIP1L and the downstream non-coding genes U6ATAC and 5 S rRNA have functional relevance for milk fat and protein yield.

It will be interesting to verify the effect of the obesity gene locus on milk energy content in humans, which might impact infant nutrition during breast feeding, and to test if the FTO region affects not only body fat but also lean mass.

## Materials and Methods

### Animals

The association study was carried out with 2,402 breeding bulls and 1,476 cows of the German Holstein population. Bulls were born between 1981 and 2003. Among bulls, a family structure of 40 full siblings and 563 half siblings was identified.

Cows descended from 296 bulls, of which 56 were breeding bulls of the analyzed bull population. The average number of cows per bull was 4.9 with a minimum of one (126 cases) to a maximum of 79 (1 case). Among cows 1,407 had finished lactation 1; 1,318 lactation 2 and 860 lactation 3. Cows that did not finish the third lactation were culled due to sickness or other reasons. Cows were managed in three herds in the Northeast of Germany [24].

### Phenotypic Data

Unless otherwise mentioned, the milk performance phenotypes we refer to, are average values of fat yield (FY), protein yield (PY), milk yield (MY), fat content (FC), and protein content (PC) for the first three lactations. Albeit it would also be interesting to analyze the association of FTO with body weight, these data have not been recorded since it is not a matter of routine in milk production.

#### Bulls

Bulls have estimated breeding values (EBVs) for their daughter performance. In our analyzed population 1.8 Million performance data records of cows contributed to breeding value estimation via a random regression model. Each bull had on average 700 daughters with performance data for the first three lactations. Performance data consists of 9 to 10 test day records per cow and lactation. EBVs of bulls refer to additive genetic variance and are highly accurate since they are based on massive daughter information, which is corrected for environmental effects across the whole population. It should be noted that in addition to the daughter performance the random regression model for estimating breeding values considers also the daughter’s pedigree. EBVs for our analyzed bull population were obtained from the center of national breeding evaluation (VIT Verden, Germany) for the production traits fat yield (FY), fat content (FC), milk yield (MY), protein content (PC) and protein yield (PY) and separated into EBVs for the first three lactations and their average.

#### Cows

Cows have own production performance data for every lactation, based on 9 to 10 test day records per lactation. For the association study in the cow population we used averaged yield deviations (YD) for the first three lactations of 860 cows that finished lactation 3. YDs give the deviation of the milk fat of a particular cow in comparison to the mean of the analyzed population after correcting for environmental effects. YDs were estimated across all cows in the population, were phenotypic data was available. Using a restricted maximum likelihood (REML) approach, YDs were estimated with the following model:
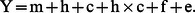



Parameters used were: Y = milk production record; m = population mean; h = fixed effect of the herd (three classes); c = fixed effect of the calving season (28 classes); h × c = interaction between herd effect and calving season; f = linear regression on age at first calving; e = random residual. From a multitude of environmental conditions that affect milk yield and composition, all known effects as herd, calving season, interaction between herd and calving season and age of first calving were considered in correction of the YDs. However, residual environmental effects are high in comparison to breeding values. With respect to “age” effects, we refer to 1st, 2nd and 3rd lactation, which differs depending on the physiological condition of the cow. During the first lactation, the mammary gland further develops, while when this endocrine system is fully established, the pathways of milk production are more effective in following lactations [25].

### Genotypic Data

#### Bulls

Genotyping of bull DNA was performed with the Illumina BovineSNP50K BeadChip [26] containing 54,001 SNPs. SNP data from this chip were subject to rigorous validation by a remapping procedure against the Btau 4.2 assembly, as suggested by [27]. In total 2,017 ambiguous SNP positions were defined as missing due to substantial deviations between the mapping strategy of the manufacturer and our own. A quality check of obtained genotypes revealed that 8,748 SNPs had to be removed, either because they failed genotyping in more than 10% of the animals (749 SNPs) or due to a minor allele frequency below 1% (7,998 SNPs). Out of 2,402 bulls, 48 were removed for low genotyping (>10% missing SNPs). FTO is located on BTA18 between 21,321,201 and 21,904,687 Mb. The SNP Hapmap49169-BTA-42663 from the Illumina BovineSNP50K BeadChip was centrally located in the FTO gene. This position was defined as the center of a 2 Mb chromosomal segment. Since SNPs are often in linkage disequilibrium with SNPs in the target gene, the FTO region was extended to 1 Mb up-and downstream of this center-SNP (20,557,461 and 22,462,625 bp). A similar partitioning of the bovine genome in chromosomal segments for association analysis was previously reported [7]. Thirty-six SNPs on the BovineSNP50K BeadChip were located in this 2 Mb region and were used for association analyses in our study.

#### Cows

The three most significant SNPs from the study in bulls (SNP1, SNP4, SNP5) and two additional SNPs (SNP2, SNP3) that contributed to the significant haplotype blocks HTB6 and HTB7 were genotyped in cows. An additional SNP was located in the DGAT1 region. Genotyping of cows was performed using allele specific KBioscience SNP-assays, as described previously [28]. Primers for SNP genotyping are listed in the supplements (Table S7 in File S1). The SNP test uses two primers for the alternative alleles and a locus specific common primer, which are used in one PCR reaction. The allele specific primers have two different primer tails that bind to complementary oligonucleotides of the PCR master mix, which are linked to two different fluorescent dyes to label the allele specific primers during PCR.

### Haplotype Inference and Block Computation

#### Bulls

Haplotype construction were carried out on a population of 2,354 sires and additional 672 German Holstein bull dams, which were genotyped with the same SNP chip on the same Illumina platform. Based on a more stringently filtered dataset (<3% missing genotypes, minor allele frequency >5%, <5% missing SNP calls), haplotypes were derived using the software Fastphase [29]. The program was run for whole chromosomes with 10 random starts (parameter -T) and 25 iterations (parameter -C). Phased genotyping data was partitioned into haplotype blocks using the solid spine algorithm implemented in the software Haploview v4.1 [30]. Brieﬂy, a block was defined if all markers within a region were in linkage disequilibrium (LD) of D’ >0.8 with the first and last marker of that region but not necessarily with each other. An exception posed two-marker blocks where a lower threshold of D’ >0.5 was used by Haploview.

#### Cows

The programme SimWalk [31] was used to infer the phase of haplotypes in the cow population. Haplotypes were inferred for HTB6 and HTB7, which gave significant results in the bull population. For the generation of haplotype phases, we used the same four SNPs that contributed to HTB6 and HTB7 in the bull population.

### Association Analysis

In a first step, association analyses were performed between SNPs or haplotypes and estimated breeding values or YDs of production performance data for milk fat yield. In a second step, all SNPs and haplotypes were tested for potential effects on other milk composition traits. Association analyses were performed with all SNPs and haplotypes with minor allele frequencies in the populations above 1%.

Four linear regression models with increasing stringency with respect to relationship between animals and to phenotypes were applied to test associations in bulls and cows (Table 1). Model 1 was the least and model 4 the most stringent model. Compared to models 1 and 2, models 3 and 4 accounted for population stratification. In addition, models 2 and 4 considered the known effect of DGAT1 on milk fat yield [17]. Therefore, in these models, the DGAT1 SNP of the BovineSNP50K (ARS-BFGL-NGS-4939) with the lowest p-value was fitted as a fixed effect to account for the allelic dosage of the DGAT1 effect, as previously described [32]. However, accounting for DGAT1 as best known major gene for milk fat synthesis affecting milk fat content is still under discussion since it is not clear, neither from a biological nor from a statistical point of view, how its variance affects the detection of other loci [25]. Therefore, a SNP or haplotype was considered significant, if one of the p-values of model 3 or 4 was <0.05. We present the result of all models to visualize the impact of population stratification and DGAT1 on the significance of association.

Association results in all four models were adjusted for multiple testing using Bonferroni [33], [34] correction (p<0.05). Bonferroni correction gives the most stringent thresholds, under the conservative assumption that all tested SNPs are independent. Although SNPs can be in LD and thus be not fully independent, it is not straightforward clear which SNP in or around a candidate gene best represents the LD to the target mutation(s) affecting the trait under analysis. Thus, we tested as many SNPs as possible in the candidate gene region, accepting the risk that some SNPs may represent repetitive information due to genetically linked marker in a specific population. The four statistical models tested, which represent different stringencies with respect to population stratification and phenotypes, were not treated as repeated tests to be accounted for in p-value correction.

For significant SNPs, effects between genotype groups were tested for significance using a Tukey-Kramer test. Haplotypes were tested for significance using the mixed model (model 4, Table 1). For generating haplotype effect plots, the phased data obtained by FASTPHASE was used. For assessing the relationship between milk fat and protein yield Pearson’s correlation coefficients conditioned for the SNP genotypes were calculated.

#### Bulls

For the association analyses, we used the software Plink v1.06 [35]. The DGAT1 SNP was added as a fixed effect in Plink using a simple 0, 1, 2 allele coding. To adjust for population stratification in the bull population an identity-by-state-similarity matrix was constructed from the genotypes of all SNPs on the BovineSNP50K BeadChip using Plink. With a pairwise population concordance test (PPC) and multi dimensional scaling (MDS), 124 significant clusters (p<0.0001) were identified, which basically served to represent the population structure as covariates in the model. By this procedure, the genomic inflation factor λ was reduced from 4.5 to a minimum value of 1.7. The deviation from λ = 1.0 is due to loci that are linked with the trait under examination and which are under selection, i.e., in our case artificial selection due to breeding for milk composition and yield traits.

#### Cows

For further validation of five significant SNP and two haplotype effects found in the bull population, association tests were performed with the same four linear models as in bulls using SAS (SAS 2008). In cows, we accounted for the population stratification by adding the father as a random effect in models 3 and 4 (PROC MIXED).

## Supporting Information

File S1 (DOCX)
