# Supplementary material for: Impact of Variation at the FTO Locus on Milk Fat Yield in Holstein Dairy Cattle
Source: PLoS One. 2013 May 15;8(5):e63406. doi: 10.1371/journal.pone.0063406 (PMC3655180; doi:10.1371/journal.pone.0063406)
Supplement: File S1 — (DOCX) [file pone.0063406.s001.docx]

**Supporting Information**

|  |  |  |  |  | **Regression model** | | | |  |
| --- | --- | --- | --- | --- | --- | --- | --- | --- | --- |
| **SNP ID** | **Position [bp]** | **A1** | **A2** | **f_A1_** | **No Adjust­ment**  **M1** | **DGAT1**  **M2** | **Populat.**  **structure**  **M3** | **DGAT1+ Population structure**  **M4** | **HTB** |
| *ARS-BFGL-NGS-4939** | 443,937 | G | A | 0.31 | 2.18e-58 | - | 7.35e-49 | - | - |
| *ARS-BFGL-NGS-111533* | 20,557,460 | A | G | 0.08 | n.s. | 0.0010 | n.s. | n.s. | - |
| *Hapmap46385-BTA-90833* | 20,670,785 | A | T | 0.03 | n.s. | n.s. | n.s. | n.s. | - |
| *Hapmap35049-BES5_Contig425_820* | 20,708,836 | A | C | 0.10 | n.s. | n.s. | n.s. | n.s. | 1 |
| *Hapmap42359-BTA-90829* | 20,733,367 | A | G | 0.36 | n.s. | n.s. | n.s. | n.s. | 1 |
| *ARS-BFGL-NGS-106934* | 20,761,751 | G | A | 0.41 | n.s. | n.s. | n.s. | n.s. | 1 |
| *Hapmap49904-BTA-19486* | 20,784,078 | A | G | 0.48 | n.s. | n.s. | n.s. | n.s. | 1 |
| *ARS-BFGL-BAC-36239* | 20,805,052 | A | G | 0.15 | n.s. | n.s. | n.s. | n.s. | 1 |
| *ARS-BFGL-NGS-43902* | 20,834,880 | G | A | 0.15 | n.s. | n.s. | n.s. | n.s. | 2 |
| *BTB-01627667* | 20,876,457 | A | G | 0.39 | n.s. | n.s. | n.s. | n.s. | 2 |
| *ARS-BFGL-BAC-36240* | 20,916,039 | A | G | 0.39 | n.s. | n.s. | n.s. | n.s. | 2 |
| *Hapmap51594-BTA-42721* | 20,977,958 | G | A | 0.41 | n.s. | 0.0708 | n.s. | n.s. | 3 |
| *Hapmap42547-BTA-42724* | 21,072,014 | G | A | 0.39 | n.s. | n.s. | n.s. | n.s. | 3 |
| *BTA-42733-no-rs* | 21,098,148 | G | A | 0.13 | n.s. | n.s. | n.s. | n.s. | 4 |
| *BTA-42735-no-rs* | 21,132,694 | A | C | 0.25 | n.s. | n.s. | n.s. | n.s. | 4 |
| *ARS-BFGL-NGS-34611* | 21,157,608 | G | A | 0.42 | n.s. | n.s. | n.s. | n.s. | 5 |
| *Hapmap36010­SCAFFOLD5029419187* | 21,196,526 | G | A | 0.39 | 0.0188 | 0.0029 | n.s. | n.s. | 5 |
| *ARS-BFGL-NGS-41145* | 21,267,130 | G | A | 0.44 | n.s. | n.s. | 0.0413 | 0.0103 | 6 |
| *Hapmap39651-BTA-42671* | 21,327,138 | C | A | 0.41 | n.s. | 0.0404 | n.s. | n.s. | 6 |
| *Hapmap49170-BTA-42666* | 21,409,626 | A | G | 0.25 | 0.0287 | n.s. | n.s. | n.s. | - |
| *ARS-BFGL-NGS-28677* | 21,464,728 | A | G | 0.41 | n.s. | n.s. | n.s. | n.s. | 7 |
| *Hapmap51449-BTA-42665* | 21,508,221 | G | A | 0.26 | 0.0083 | 0.0002 | 0.0563 | 0.0129 | 7 |
| *Hapmap49169-BTA-42663* | 21,689,812 | G | A | 0.36 | n.s. | 0.0256 | n.s. | n.s. | - |
| *ARS-BFGL-NGS-17185* | 21,729,770 | G | A | 0.35 | 0.0088 | 8.06e-05 | n.s. | 0.0172 | - |
| *BTA-42645-no-rs* | 21,874,572 | C | A | 0.30 | n.s. | n.s. | n.s. | n.s. | 8 |
| *ARS-BFGL-NGS-13888* | 21,907,404 | G | A | 0.40 | n.s. | n.s. | n.s. | 0.0496 | 8 |
| *ARS-BFGL-NGS-23701* | 21,962,228 | A | C | 0.31 | n.s. | n.s. | n.s. | n.s. | 8 |
| *ARS-BFGL-NGS-19178* | 21,991,700 | A | G | 0.28 | n.s. | 0.0629 | 0.0666 | 0.0279 | - |
| *ARS-BFGL-BAC-36256* | 22,068,361 | A | G | 0.30 | n.s. | n.s. | n.s. | n.s. | 9 |
| *Hapmap52930-rs29019965* | 22,113,180 | G | A | 0.45 | 0.0067 | 0.0145 | n.s. | n.s. | 9 |
| *ARS-BFGL-NGS-24172* | 22,144,501 | A | C | 0.22 | n.s. | n.s. | n.s. | n.s. | - |
| *UA-IFASA-3993* | 22,242,400 | A | G | 0.29 | n.s. | n.s. | n.s. | n.s. | - |
| *ARS-BFGL-NGS-25646* | 22,273,228 | G | A | 0.25 | n.s. | n.s. | n.s. | n.s. | - |
| *BTA-31073-no-rs* | 22,340,688 | A | G | 0.44 | n.s. | n.s. | n.s. | n.s. | 10 |
| *Hapmap51823-BTA-31074* | 22,409,465 | A | G | 0.35 | n.s. | n.s. | n.s. | n.s. | 10 |
| *BTA-04489-rs29014848* | 22,430,409 | A | G | 0.07 | n.s. | n.s. | n.s. | n.s. | 10 |
| *ARS-BFGL-NGS-1042* | 22,462,624 | A | C | 0.23 | 0.0465 | n.s. | n.s. | n.s. | - |

**Table S1.** Results of association analyses of all SNPs within the 2 Mb FTO candidate region on chromosome BTA18.

*= DGAT1-SNP on chromosome 14. Grey shaded cells belong to SNPs directly located within the FTO gene. Shown are p-values resulting from association of genotypes with breeding values averaged over lactations one to three with model 1 to 4 (M1-M4); HTB = index of haplotype blocks that have been found for SNPs. f_A1_ = population frequency of the minor allele. Positions refer to the Btau 4.2 assembly. P-values > 0.1 are labeled ”n.s.”.

**Table S2.** Haplotype blocks in the FTO region of the analyzed HF bull population.

| **Block ID** | **Block size [kb]** | **D‘** | **SNP ID** | **Haplotypes** | **f** |
| --- | --- | --- | --- | --- | --- |
| HTB1 | 96.22 | 0.97 | *Hapmap35049-BES5_Contig425_820*  *Hapmap42359-BTA-90829*  *ARS-BFGL-NGS-106934*  *Hapmap49904-BTA-19486*  *ARS-BFGL-BAC-36239* | CAAAG  CGGGG  CGGGA  CGAAG  CGAGG  AGGGG | 0.359  0.169  0.147  0.121  0.105  0.091 |
| HTB2 | 81.16 | 0.99 | *ARS-BFGL-NGS-43902*  *BTB-01627667*  *ARS-BFGL-BAC-36240* | AGG  AAA  GGG | 0.463  0.390  0.147 |
| HTB3 | 94.06 | 0.72 | *Hapmap51594-BTA-42721*  *Hapmap42547-BTA-42724* | GA  AG  AA  GG | 0.362  0.343  0.251  0.043 |
| HTB4 | 34.55 | 0.96 | *BTA-42733-no-rs*  *BTA-42735-no-rs* | AC  AA  GA | 0.735  0.131  0.130 |
| HTB5 | 38.92 | 0.59 | *ARS-BFGL-NGS-34611*  *Hapmap36010-SCAFFOLD50294_19187* | AA  GG  GA  AG | 0.486  0.300  0.123  0.092 |
| HTB6 | 60.00 | 0.86 | *ARS-BFGL-NGS-41145**  *Hapmap39651-BTA-42671* | AA  GC  GA  AC | 0.523  0.373  0.072  0.032 |
| HTB7 | 43.49 | 0.98 | *ARS-BFGL-NGS-28677*  *Hapmap51449-BTA-42665** | AA  GA  GG | 0.407  0.335  0.257 |
| HTB8 | 87.66 | 0.65 | *BTA-42645-no-rs*  *ARS-BFGL-NGS-13888**  *ARS-BFGL-NGS-23701* | AAC  AGA  CAC  CGC  CGA | 0.404  0.293  0.194  0.086  0.018 |
| HTB9 | 44.82 | 0.83 | *ARS-BFGL-BAC-36256*  *Hapmap52930-rs29019965* | GA  AG  GG  AA | 0.522  0.271  0.180  0.027 |
| HTB10 | 89.72 | 0.99 | *BTA-31073-no-rs*  *Hapmap51823-BTA-31074*  *BTA-04489-rs29014848* | GGG  AAG  AGG  AAA | 0.561  0.285  0.083  0.069 |

Numbers of haplotype blocks are given in order of their occurrence in the FTO region on chromosome 18.

* = SNPs significantly (p < 0.05) associated with the average EBV for fat yield of lactations one to three; D’ = average linkage disequilibrium within spine block SNPs (see methods); f = haplotype frequency.

**Table S3.** Phenotypic means and frequencies of SNP genotype classes.

| **Popu-lation** | **SNP number** | **SNP ID** | **Genotype** | **Absolute Frequency** | **Relative Frequency** | **µ ± se (FY)** | **Median** |
| --- | --- | --- | --- | --- | --- | --- | --- |
| A | 1 | *ARS-BFGL-NGS-41145* | GG  GA  AA | 476  1159  711 | 0.203  0.493  0.303 | 21.76±1.10  19.82±0.72  18.84±0.85 | 22.45  20.00  19.00 |
|  | 2 | *Hapmap39651-BTA-42671* | CC  CA  AA | 372  1144  829 | 0.158  0.487  0.353 | 21.84±1.25  20.04±0.73  18.87±0.79 | 21.80  20.50  19.00 |
|  | 3 | *ARS-BFGL-NGS-28677* | AA  GA  GG | 395  1113  840 | 0.168  0.474  0.358 | 22.84±1.16  19.67±0.71  18.89±0.85 | 23.30  19.10  19.50 |
|  | 4 | *Hapmap51449-BTA-42665* | GG  GA  AA | 158  896  1293 | 0.067  0.381  0.550 | 15.70±1.82  18.59±0.78  21.41±0.67 | 18.20  18.45  21.60 |
|  | 5 | *ARS-BFGL-NGS-17185* | GG  GA  AA | 363  940  1044 | 0.154  0.400  0.444 | 22.52 ± 1.21  21.20 ± 0.77  17.92 ± 0.75 | 18.20  18.45  21.60 |
|  | 6 | *ARS-BFGL-NGS-13888* | GG  GA  AA | 390  1084  873 | 0.166  0.462  0.372 | 17.66 ± 1.16  19.95 ± 0.71  20.89 ± 0.84 | 21.60  18.45  18.20 |
|  | 7 | *ARS-BFGL-NGS-19178* | AA  GA  GG | 191  948  1210 | 0.08  0.404  0.515 | 15.42 ± 1.67  19.85 ± 0.77  20.69 ± 0.69 | 17.00  19.90  20.85 |
| B | 1 | *ARS-BFGL-NGS-41145* | GG  GA  AA | 163  396  276 | 0.202  0.491  0.307 | 10.31 ± 3.24  2.99 ± 2.02  0.84 ± 2.65 | 9.43  4.88  3.41 |
|  | 2 | *Hapmap39651-BTA-42671* | CC  CA  AA | 151  435  267 | 0.177  0.510  0.313 | 9.39±3.22  7.29±2.01  -2.43±2.59 | 6.79  8.21  1.50 |
|  | 3 | *ARS-BFGL-NGS-28677* | AA  GA  GG | 171  427  213 | 0.211  0.527  0.263 | 1.74±3.18  4.02±2.00  5.28±2.68 | 1.89  5.98  4.69 |
|  | 4 | *Hapmap51449-BTA-42665* | GG  GA  AA | 72  271  513 | 0.084  0.317  0.599 | -5.01±4.59  3.04±2.48  6.84±1.89 | -6.39  5.69  6.86 |
|  | 5 | *ARS-BFGL-NGS-17185* | GG  GA  AA | 98  341  359 | 0.123  0.427  0.450 | 3.74±4.48  5.07±2.16  3.30±2.14 | 10.16  7.11  3.15 |

Shown are genotype frequencies and phenotypic means with standard error (µ ± se) of genotype classes belonging to SNPs significantly (p < 0.05) associated with the average EBV of bulls (**A**) or yield deviation of cows (**B**) for fat yield (FY) over lactations one to three. µ is given in kilogram fat as EBV units in bulls and YD units in cows. Since the correction for environmental effects differs between EBV and YD, the units are not identical.

**Table S4.** Results of SNP association analysis for additional milk traits.

|  |  |  |  |  |  |  | **Regression model** | | | | | |
| --- | --- | --- | --- | --- | --- | --- | --- | --- | --- | --- | --- | --- |
| **Popu-lation** | **Trait** | **SNP number** | **SNP ID** | **A1** | **A2** | **f_A1_** | **No Adjust­ment** | **DGAT1** | **Population structure** | **DGAT1 + Population structure** | **β** | ***a*** |
|  |  |  |  |  |  |  | **Model 1** | **Model 2** | **Model 3** | **Model 4** | **Model 4** | **Model4** |
| A | PY | 1 | *ARS-BFGL-NGS-41145* | G | A | 0.45 | 0.9991 | n.s. | 0.0305 | 0.0319 | 1.79 | 1.59 |
|  |  | 2 | *Hapmap39651-BTA-42671* | C | A | 0.41 | 0.0333 | n.s. | 0.0214 | n.s. | 1.42 | 1.76 |
|  |  | 3 | *ARS-BFGL-NGS-28677* | A | G | 0.41 | n.s. | n.s. | n.s. | n.s. | 0.66 | 0.08 |
|  |  | 4 | *Hapmap51449-BTA-42665* | G | A | 0.26 | 0.0001 | 0.0004 | 0.0123 | 0.0108 | -2.17 | 2.63 |
|  |  | 5 | *ARS-BFGL-NGS-17185* | G | A | 0.35 | 0.0241 | n.s. | n.s. | n.s. | 1.32 | 1.73 |
| A | MY | 1 | *ARS-BFGL-NGS-41145* |  |  |  | 0.0069 | 0.0435 | 0.0050 | 0.0023 | 68.68 | 67.40 |
|  |  | 2 | *Hapmap39651-BTA-42671* |  |  |  | n.s. | n.s. | n.s. | n.s. | 37.32 | 46.50 |
|  |  | 3 | *ARS-BFGL-NGS-28677* |  |  |  | n.s. | n.s. | n.s. | n.s. | 18.53 | 7.55 |
|  |  |  | *Hapmap51449-BTA-42665* |  |  |  | 0.0149 | 0.0684 | n.s. | 0.0746 | -59.47 | 53.00 |
|  |  |  | *ARS-BFGL-NGS-17185* |  |  |  | n.s. | n.s. | n.s. | n.s. | 35.92 | 46.25 |
| B | FC | 1 | *ARS-BFGL-NGS-41145* | G | A | 0.45 | 0.0016 | 0.0082 | 0.0113 | 0.0534 | 0.032 | 0.070 |
|  |  | 2 | *Hapmap39651-BTA-42671* | C | A | 0.43 | 0.0036 | 0.0117 | 0.0142 | n.s. | 0.033 | 0.061 |
|  |  | 3 | *ARS-BFGL-NGS-28677* | A | G | 0.47 | n.s. | n.s. | n.s. | n.s. | -0.004 | -0.006 |
|  |  | 4 | *Hapmap51449-BTA-42665* | G | A | 0.24 | 0.0178 | 0.0159 | 0.0330 | 0.0447 | -0.044 | -0.069 |
|  |  | 5 | *ARS-BFGL-NGS-17185* | G | A | 0.34 | n.s. | n.s. | n.s. | n.s. | 0.019 | 0.011 |
| B | PC | 1 | *ARS-BFGL-NGS-41145* |  |  |  | 0.0384 | n.s. | 0.0829 | n.s. | 0.010 | 0.018 |
|  |  | 2 | *Hapmap39651-BTA-42671* |  |  |  | n.s. | n.s. | n.s. | n.s. | 0.006 | 0.018 |
|  |  | 3 | *ARS-BFGL-NGS-28677* |  |  |  | n.s. | n.s. | n.s. | n.s. | 0.008 | 0.005 |
|  |  | 4 |  |  |  |  | 0.0335 | 0.0453 | 0.0373 | 0.0654 | -0.020 | -0.025 |
|  |  | 5 |  |  |  |  | n.s. | n.s. | n.s. | n.s. | 0.006 | 0.006 |

Shown are p-values for SNPs in the 2Mb FTO region on chromosome BTA18 in the bull- (**A**) and cow population (**B**) that were beforehand significantly associated with the average EBV for fat yield over lactations one to three in the bull population after applying model 4. P-values for model 1-3 are additionally shown for comparison. Positions refer to the Btau 4.2 assembly. f_A1_ = population frequency of the minor allele; β = effect size, giving the per minor allele change of the trait; *a* = additive effect, defined as the deviation of the phenotypic value (here breeding value) of the homozygous genotype classes from the midpoint of the population. α and β are given in kilogram fat as EBV units in bulls and YD units in cows. Since the correction for environmental effects differs between EBV and YD, the units are not identical.

**Table S5.** Results of haplotype association analysis for additional milk traits.

|  |  |  |  |  | **Regression model** | | | | |  |
| --- | --- | --- | --- | --- | --- | --- | --- | --- | --- | --- |
| **Population** | **Trait** | **Block ID** | **HPT** | **f_hpt_** | **No Adjustment** | **DGAT1** | **Population structure** | **DGAT1 + Population structure** | **β** | **µ ± se (trait)** |
|  |  |  |  |  | **Model 1** | **Model2** | **Model3** | **Model4** | **Model4** |  |
| A | PY | HTB6 | AA | 0.52 | 0.0005 | 0.0045 | 0.0070 | 0.0070 | -2.00 | 20.80±0.37 |
|  |  |  | AC | 0.03 | 0.0008 | 0.0071 | n.s. | n.s. | 2.22 | 28.81±1.61 |
|  |  | HTB7 | GA | 0.03 | 0.0005 | 0.0253 | n.s. | n.s. | 1.15 | 23.64±0.48 |
|  |  |  | GG | 0.26 | 0.0001 | 0.0005 | 0.0126 | 0.0126 | -2.17 | 19.78±0.54 |
| A | PC | HTB6 | GA | 0.08 | 0.0928 | 0.0294 | 0.0105 | 0.0105 | -0.02 | -0.05±0.01 |
|  |  |  | AC | 0.03 | n.s. | 0.0476 | n.s. | n.s. | 0.02 | -0.00±0.01 |
| A | FC | HTB6 | GA | 0.08 | n.s. | 0.0317 | n.s. | n.s. | -0.03 | -0.15±0.02 |
| A | MY | HTB6 | AA | 0.52 | 0.0002 | 0.0055 | 0.0018 | 0.0018 | -69.60 | 679.62±12.10 |
|  |  |  | GA | 0.08 | 0.0110 | 0.0049 | 0.0068 | 0.0068 | 120.00 | 841.72±31.24 |
|  |  | HTB7 | GA | 0.34 | 0.0082 | n.s. | n.s. | n.s. | 30.00 | 766.35±15.85 |
|  |  |  | GG | 0.26 | 0.0210 | 0.0988 | n.s. | n.s. | -58.10 | 666.33±18.04 |
| B | FC | HTB6 | AA | 0.45 | 0.0010 | 0.0076 | 0.0087 | n.s. | -0.03 | -0.04±0.04 |
|  |  |  | GG | 0.21 | 0.0042 | 0.0040 | 0.0170 | 0.0335 | -0.05 | -0.05±0.02 |
| B | PC | HTB6 | GC | 0.31 | 0.0181 | 0.0410 | n.s. | n.s. | 0.009 | 0.01±0.0 |
|  |  | HTB7 | GG | 0.21 | 0.0099 | 0.0248 | 0.0186 | 0.0550 | -0.02 | -0.02±0.00 |

Shown are p-values for haplotypes of the significant haplotype blocks in the 2Mb FTO region on chromosome BTA18 that were significantly associated with the average EBV for fat yield over lactations one to three in the bull -(**A**) and cow population (**B**) after applying model 4. P-values for models 1-3 are additionally shown for comparison. P-values > 0.1 are labeled “n.s.”. β = effect size (regression coeﬃcient) giving the per haplotype change of the trait. µ±se denotes the phenotypic mean with standard error of the haplotype class based on EBVs in bulls and YDs in cows. HPT = haplotype; f_hpt_ = haplotype population frequency; **PY** = protein yield; **PC** = protein content; **FC** = fat content; **MY** = milk yield. β and µ are given in kilogram fat as EBV units in bulls and YD units in cows. Since the correction for environmental effects differs between EBV and YD, the units are not identical.

**Table S6.** Linkage disequilibrium between significant SNPs in the FTO region.

| **Population** | **SNP ID** | **SNP number** | **SNP2** | **SNP3** | **SNP4** | **SNP5** |
| --- | --- | --- | --- | --- | --- | --- |
| A | *ARS-BFGL-NGS-41145* | 1 | **0.858** / **0.625** | 0.036 / 0.001 | **0.759*** / **0.161** | 0.094 / 0.006 |
|  | *Hapmap39651-BTA-42671* | 2 | 1.000 | 0.168 / 0.013 | **0.880** / **0.184** | 0.060 / 0.003 |
|  | *ARS-BFGL-NGS-28677* | 3 |  | 1.000 | **0.983** / **0.233** | 0.048 / 0.002 |
|  | *Hapmap51449-BTA-42665* | 4 |  |  | 1.000 | **0.869** / **0.140** |
|  | *ARS-BFGL-NGS-17185* | 5 |  |  |  | 1.000 |
| B | *ARS-BFGL-NGS-41145* | 1 | **0.512** | 0.0002 | **0.147** | 5.43e-05 |
|  | *Hapmap39651-BTA-42671* | 2 | 1.000 | 0.003 | **0.132** | 0.018 |
|  | *ARS-BFGL-NGS-28677* | 3 |  | 1.000 | **0.199** | 0.004 |
|  | *Hapmap51449-BTA-42665* | 4 |  |  | 1.000 | 0.066 |
|  | *ARS-BFGL-NGS-17185* | 5 |  |  |  | 1.000 |

Shown are and D’ and r^2^ values, separated by “/” for bulls (**A**) and r^2^ values for cows (**B**) for SNPs in the FTO region that are significantly associated with

the average EBV (bulls) and/or yield deviations (cows) for fat yield over lactations one to three. SNP pairs in high linkage disequilibrium are indicated in bold face.

*For the linkage between SNP1 and SNP4 see also Figure 3.

**Table S7.** Primer sequences for SNP genotyping in the cow population.

| **SNP ID** | **SNP number** | **Primer Type** | **Primer Sequence** |
| --- | --- | --- | --- |
| *ARS-BFGL-NGS-41145* | 1 | Allele T  Allele C  Common Primer | GAAGGTGACCAAGTTCATGCTGTAATCATAGTTCCATAGAGGGCCA**T**  GAAGGTCGGAGTCAACGGATTAATCATAGTTCCATAGAGGGCCA**C**  TCAGAAGAGAGATTAGCAAAGGGAGTTAT |
| *Hapmap39651-BTA-42671* | 2 | Allele T  Allele G  Common Primer | GAAGGTGACCAAGTTCATGCTCGTCACTGTCTTTATGAAACCG**T**  GAAGGTCGGAGTCAACGGATTCGTCACTGTCTTTATGAAACCG**G**  CACATCACTGGACATCAGAAAAAGGAAAT |
| *ARS-BFGL-NGS-28677* | 3 | Allele G  Allele A  Common Primer | GAAGGTGACCAAGTTCATGCTATACCTGCCTGGCCCCC**G**  GAAGGTCGGAGTCAACGGATTGATACCTGCCTGGCCCCC**A**  GCCTATGGGCTAGAGTGGGGTT |
| *Hapmap51449-BTA-42665* | 4 | Allele A  Allele G  Common Primer | GAAGGTGACCAAGTTCATGCTAATGAATACAAGTGTAGTTCAACTTCTCT**A**  GAAGGTCGGAGTCAACGGATTGAATACAAGTGTAGTTCAACTTCTCT**G**  CCCCTTGTAATAAATAATGTTGTTAAACCT |
| *ARS-BFGL-NGS-17185* | 5 | Allele A  Allele G  Common  Primer | GAAGGTGACCAAGTTCATGCTTAAACAGAGGCCGTAGCCCC**A**  GAAGGTCGGAGTCAACGGATTAACAGAGGCCGTAGCCCC**G**  TGTCAGAAATGCCAGCCTAGCTGAA |

The allele specific SNP test uses two allele specific primers for the alternative alleles and a locus specific common primer. The allele specific primers have two different primer tails (the SNP allele is indicated in boldface) that bind to complementary oligonucleotides of the PCR master mix, which are linked to two different fluorescent dyes to label the allele specific primers during PCR (see [[1](#_ENREF_1)])

References

1. Kreuzer S, Reissmann M, Brockmann GA (2013) New fast and cost-effective gene test to get the ETEC F18 receptor status in pigs. Vet Microbiol 163: 392-394.
